# The Importance of Cardiac T2* Magnetic Resonance Imaging for Monitoring Cardiac Siderosis in Thalassemia Major Patients

**DOI:** 10.3390/tomography7020012

**Published:** 2021-04-18

**Authors:** Narumol Chaosuwannakit, Pattarapong Makarawate, Chinnadol Wanitpongpun

**Affiliations:** 1 Radiology Department, Faculty of Medicine, Khon Kaen University, Khon Kaen 40000, Thailand; 2 Internal Medicine Department, Faculty of Medicine, Khon Kaen University, Khon Kaen 40000, Thailand; nchaosuw@gmail.com (P.M.); naruch@kku.ac.th (C.W.)

**Keywords:** Cardiac T2*, magnetic resonance imaging, thalassemia, iron overload, iron chelation therapy

## Abstract

*Objective:* Cardiac T2* magnetic resonance imaging (MRI) has recently attracted considerable attention as a non-invasive method for detecting iron overload in various organs in thalassemia major patients. This study aimed to identify the prevalence of cardiac siderosis in thalassemia major patients and evaluate cardiac T2* MRI for monitoring cardiac siderosis before and after patients receive iron chelation therapy and its relation to serum ferritin, left ventricular ejection fraction, and liver iron concentration. The information gathered would be used for the direct monitoring, detection, and treatment of complications early on. *Methods:* A total of 119 thalassemia major patients were recruited in the present study. The cardiac T2* MRI was compared to serum ferritin levels, liver iron concentration (LIC), and left ventricular ejection fraction. All patients were classified into four groups based on their cardiac siderosis as having normal, marginal, mild to moderate, or severe cardiac iron overload. At the follow-up at years one, three, and five, the cardiac T2* MRI, LIC, serum ferritin, and left ventricular ejection fraction (LVEF) were determined. *Results:* The prevalence of cardiac siderosis with cardiac T2* MRI ≤ 25 ms was 17.6% (n = 21). There was no correlation between cardiac T2* MRI and serum ferritin, liver iron concentration, and LVEF (*p* = 0.39, 0.54, and 0.09, respectively). During one year to five years’ follow-up periods, cardiac T2* MRI (ms) in patients with severe cardiac siderosis had significantly improved from 8.5 ± 1.49 at baseline to 33.9 ± 1.9 at five years (*p* < 0.0001). Patients with severe, mild-moderate, marginal, and no cardiac siderosis had median LIC (mg/g dw) of 23.9 ± 6.5, 21.6 ± 13.3, 25.3 ± 7.7, and 19.9 ± 5.5 at baseline, respectively. *Conclusions:* This study supports the use of cardiac T2* MRI to monitor cardiac iron overload in patients who have had multiple blood transfusions. Early diagnosis and treatment of patients at risk of cardiac siderosis is a reasonable method of reducing the substantial cardiac mortality burden associated with myocardial siderosis. Cardiac T2* MRI is the best test that can identify at-risk patients who can be managed with optimization of their chelation therapy.

## 1. Introduction

Patients with thalassemia major typically require regular blood transfusions beginning in childhood. Despite iron chelation therapy, heart failure due to post-transfusion iron overload is still the leading cause of death in this disease [1,2,3]. Chelation therapy has been shown to be effective in eliminating cardiac iron and in improving survival. Late in the course of the disease, life-threatening complications such as heart failure and arrhythmias develop. Cardiomyopathy is more common in patients with transfusion-dependent thalassemia (TDT), while pulmonary hypertension is more common in non-transfusion-dependent thalassemia (NTDT), particularly after splenectomy. If intense chelation is undertaken early, dilated cardiomyopathy induced by severe myocardial siderosis may be reversed, but clinical diagnosis is frequently delayed due to the late onset of symptoms. There are few predictors of heart function deterioration. Serum ferritin and hepatic iron accumulation have poor cardiac function correlations, and elevated brain-natriuretic peptide appears only late in the disease’s development [4,5,6]. Once clinically diagnosed heart failure is established, cardiac function can rapidly deteriorate and become fatal. Evaluations of ventricular function, such as changes in ejection fraction over time, have been recommended in the case of thalassemia. However, they identify patients at a late stage. In the absence of myocardial iron loading, impaired cardiac function could be obscured by supranormal left ventricular function in this patient population [6,7].

Attributed to the reason that iron deposits shorten T1, T2, and T2*, direct assessment of myocardial iron-utilizing magnetic resonance relaxation (MR) has recently become essential. Magnetic resonance imaging (MRI) techniques that identify and quantify iron in tissues could assess the amount of hepatic and cardiac iron. In a previous study, T2*sequences were used to identify and reproduce techniques for measuring myocardial iron concentration. If cardiac iron overload is detected, patients at risk of heart failure will be determined before becoming symptomatic [8,9,10].

Cardiac T2* MRI has recently allowed for more accurate measurement of iron overload in the heart, allowing a better guide for managing iron chelation therapy with single or multiple chelators [8,9]. For many years, Thai physicians have been using a standardized and validated analytical method to measure cardiac and hepatic iron overload using a specific T2* MRI sequence [11,12]. They implemented a specific T2* MRI sequence to provide a comprehensive measurement of hepatic and myocardial iron overload in a single MRI study. Nevertheless, in thalassemia major patients, the study of extensive cardiac iron status monitoring is still deficient. This study aimed to determine the prevalence of cardiac siderosis among thalassemia major patients in Northeastern Thailand and evaluate the value of using T2* MRI for monitoring cardiac and liver iron overload. Another purpose was to assess the correlation between cardiac T2* MRI and liver iron concentration (LIC), serum ferritin, and left ventricular ejection fraction.

## 2. Materials and Methods

### 2.1. Patients

We accomplished a retrospective cohort study to evaluate the prevalence of cardiac iron overload in thalassemia major patients who attended the Adult Hematology Clinic at Khon Kaen University, Thailand. The patients who underwent cardiac T2* MRI and had complete medical records were recruited in the study between January 2016 and December 2020. Cardiac T2*magnetic resonance imaging (MRI) was used to evaluate myocardial iron overload, and echocardiography was used to assess cardiac function. There were 54 men and 65 women with a mean age of 29.9 ± 19.6 years at the time of their first baseline MRI. All of the MRI scans were performed in the radiology department of Khon Kaen University. T2* ≤ 25 milliseconds (ms) routinely prompted intensifying the iron chelation therapy (increasing doses or introducing combination therapy).

### 2.2. Magnetic Resonance Imaging Interpretation

The images were obtained on a 1.5-T Avanto system (Siemens Medical Solutions, Erlangen, Germany) using a cardiac phased-array coil and a standard protocol system. The imaging acquisition began with a cardiac assessment followed by a liver evaluation. The optimized black-blood sequences were used to scan each patient. A specific double inversion recovery pre-pulse WAS used in the black-blood technique to null the blood signal in the heart chamber. For the black blood techniques, a single mid-ventricular short-axis slice was obtained with slice thicknesses of 6 and 10 mm. The imaging parameters were 19 ms TR, 8 echo times, a 128 × 256 matrix, and a 40 cm field of view (FOV), yielding a voxel scale of 3.1 × 1.6 × 10 mm^3^ the inversion time (TI) set to suppress the blood signal. A multi-echo fast gradient-recalled echo sequence was acquired within a single breath-hold during an MRI scan of the liver at the mid-hepatic slice. The imaging parameters were 80 msec repetition time (TR), 20 echo times (1.1–16.3 msec with 0.8 msec increments), 10 mm slice thickness, 20-degree flip angle, and 40 cm field-of-view (FOV). The total scan time is about 10–15 min. The images were analyzed locally and then sent to the reference site in DICOM format for processing. Both T2* analyses were carried out independently at the site using custom software [12,13]. Regions of interest (ROIs) were manually determined at the interventricular septum, as in the standard method, using prior knowledge of a cardiac T2* MRI map to avoid susceptibility artifacts and a partial volume effect at the interventricular septum’s edge. To assess liver iron overload, which was determined by manually defining the ROIs from the whole region of the liver after excluding the major vessels [12,13], the analyses were carried out independently by a radiologist with more than ten years of cardiac MR analysis expertise. All of the models in this study’s T2* results were reported using both mean and median values.

To evaluate the liver iron concentration (LIC), the single trans-axial slice through the liver’s central was imaged at eight echo times to measure the liver iron concentration (2.3–16 ms). Without cardiac gating, the TR was set to 200 ms. The signal intensity analysis was done away from the large central vessels in the liver’s periphery. The results regarding the severity of cardiac siderosis and liver iron overload followed those of previous studies [12,13]. The patients were allocated into four categories depending on the severity of cardiac siderosis; normal > 25 msec, marginal 20–25 msec, mild to moderate 10–20 msec, and severe ≤ 10 msec. Normal, mild, moderate, and severe liver iron concentrations were determined using cut-off values of LIC as follows: normal ≤ 3 mg Fe/g dw, mild 3–7 mg/g dw, moderate 7–15 mg/g dw, and severe ≥ 15 mg/g dw [12,13].

### 2.3. Echocardiography

The cardiologists performed the echocardiography, which included two-dimensional, M mode, and Doppler echocardiography at rest. The measurements of the left ventricle were calculated and graded according to the methods used in previous studies [6,14,15]. The left ventricular ejection fraction (LVEF) was determined using a modified Simpson’s rule [6,14,15].

### 2.4. Statistical Analysis

Statistical analysis was performed using commercially available software (SPSS Inc., version 16.0, Chicago, IL, USA, and STATA 15 (StataCorp, College Station, Texas)). All patient demographics are presented based on the patient’s first scan. Kolmogorov—Smirnov tests revealed that serum ferritin and left ventricular ejection fraction distributions were not significantly different from normal, so they are presented as mean and SD. Pearson’s correlation equation was used to determine the correlation between different variables. Repeated measure ANOVA with Greenhouse—Geisser was selected to evaluate the difference between the two datasets. Statistical significance was defined as a *p*-value of less than 0.05.

## 3. Results

One hundred nineteen patients were included in the study. The baseline patients’ characteristics with normal, marginal, mild to moderate, and severe cardiac siderosis are summarized in Table 1. There was no significant difference in age, sex, thalassemia type, mean pre-transfusion hemoglobin level, mean baseline ferritin levels, liver iron concentration, and left ventricular ejection fraction among the four groups at baseline.

The prevalence of cardiac siderosis with cardiac T2* MRI ≤ 25 ms was 17.6% (n = 21). Patients with severe cardiac siderosis had a mean cardiac T2* MRI = 8.5 ± 1.5 ms at baseline, which consistently improved over the treatment course of 5 years. At years 3 and 5, significant improvement was noted when cardiac T2* MRI reached a level of 30.3 ± 6.1 ms and 33.9 ± 1.9 ms, respectively (*p* < 0.0001) (Table 2). Those patients who at baseline had a mild to moderate cardiac siderosis with a mean cardiac T2* MRI of 15.8 ± 3.4 ms achieved significant improvement by year 3 when the level reached 34.2 ± 4.6 ms (*p* < 0.0001). Patients with baseline marginal cardiac siderosis with mean cardiac T2* MRI of 23.2 ± 1.2 ms achieved significant improvement by year 1 when the level reached 31.8 ± 5.8 ms (*p* < 0.0001). Patients with no cardiac siderosis had stable levels of 43.1 ± 7.2 ms at baseline and 43.6 ± 5.6 ms at year 5 (Table 2). Only one of our patients had a clinical cardiac failure (LVEF of 54% and cardiac T2* MRI of 6.9 ms).

At their baseline MRI, 21 of the 119 patients had a cardiac T2* MRI ≤ of 25 ms. For 16 of these patients, iron chelation therapy was changed: combined therapy was adopted for nine patients (combined regimens of deferiprone and deferoxamine), an increased dosage of the chelating agent used as monotherapy was given to five patients, and the agent used as monotherapy was changed for two patients.

**Figure 1:**
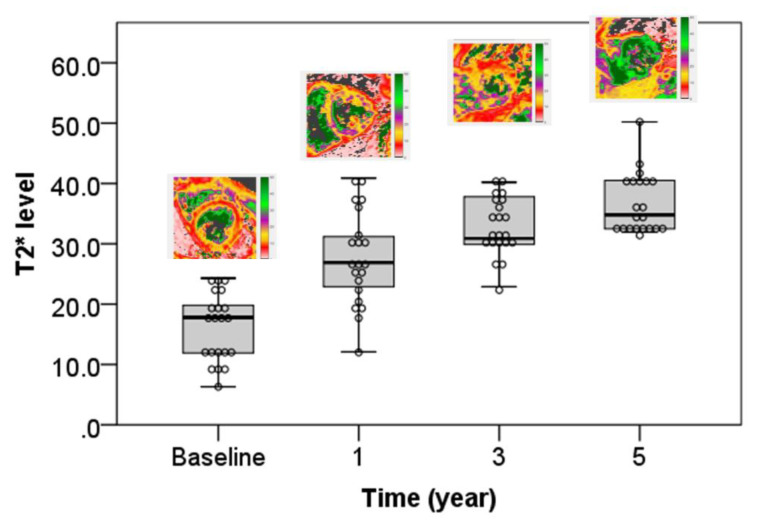
Significant improvement and increased cardiac T2* value (msec) at baseline, one year, three years, and five years.

**Figure 2:**
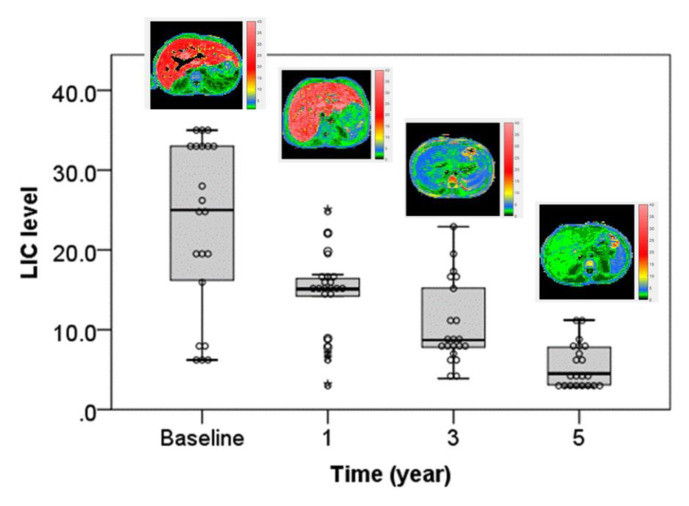
Significant improvement and decreased liver iron concentration (LIC) value (mg/g dw) at baseline, one year, three years, and five years.

An analysis of the change in T2* values over time showed a significant improvement in cardiac T2* MRI (Table 3, Figure 1). A significant reduction in liver iron concentration over time was also demonstrated (Table 3, Figure 2).

Patients were given different iron chelation regimens to optimize chelation effectiveness during this retrospective study. The majority of the patients with cardiac siderosis were on desferrioxamine (DFO) chelation (52.4%), and most of the patients without cardiac siderosis received deferasirox (DFX) iron chelation therapy (50%) (Table 4). Iron clearance from the heart has been shown to have slow kinetics. To assess the efficacy of cardiac iron chelators, prospective longitudinal studies are required. Patients with cardiac siderosis received a significantly lower volume of red cell transfusion relative to the patients without cardiac siderosis (Table 4).

**Figure 3:**
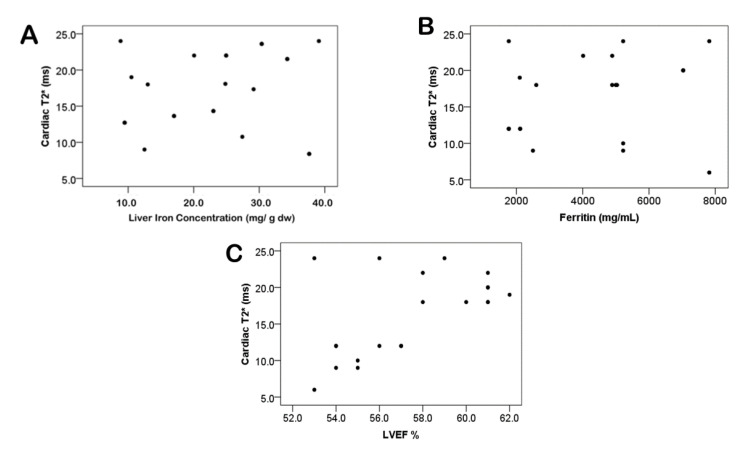
No significant correlation between cardiac T2* MRI and liver iron concentration (**A**) *p* = 0.39, serum ferritin (**B**) *p* = 0.54, and left ventricular ejection fraction (**C**) *p* = 0.09.

No significant correlation was detected between cardiac T2* MRI and liver iron concentration, serum ferritin, and LVEF (Figure 3). During one year, three years, and five years’ follow-up periods, cardiac T2* MRI in patients with severe cardiac siderosis had significantly improved from 8.5 ± 1.49 ms at baseline to 17.05 ± 3.43 ms, 30.33 ± 6.1 ms, and 33.9 ± 1.9 ms (*p* < 0.0001), respectively. Patients with severe, mild-moderate, marginal, and no cardiac siderosis had mean LIC values (mg/g dw) of 23.9 ± 6.5, 21.6 ± 13.3, 25.3 ± 7.7, and 19.9 ± 5.5 at baseline, respectively, whereas LVEF (%) was 58.3 ± 11.1, 61.9 ± 13.6, 59.5 ± 13.2, and 63.6 ± 14.5, respectively.

## 4. Discussion

In previous studies, myocardial iron deposition was found to be unrelated to serum ferritin, liver iron concentration, and even myocardial biopsy [8,9,12,16]. However, as seen in recent studies in patients with thalassemia major (TM), cardiac T2* MRI tends to comply with iron measures by myocardial biopsy and post-mortem hearts [16,17,18]. In patients with transfusion-dependent thalassemia, non-invasive serial monitoring of cardiac T2* MRI for cardiac siderosis is now practical. In TM patients, we present the sequence of annual cardiac T2* MRI measurements. Patients were given various iron chelation regimens to improve chelation efficacy during our study. We observed cardiac T2* MRI improvement from the first year in all cardiac siderosis subgroups and normalization at three years in patients with severe cardiac siderosis (Table 2).

While sequential heart function quantification recognizes patients at increased risk of cardiac mortality [6], identifying high-risk patients early, before cardiomyopathy develops, and treating them with appropriate therapy would be preferred [2,3,5,8]. Cardiac siderosis (T2* of ≤ 25 ms) in this retrospective cohort study was 17.6%. This result was much lower than those previously reported by various studies that reported a prevalence of up to 86% [14,15,16]. The TM major patients in the present study had a 3.4% prevalence of severe cardiac siderosis. Previous studies have reported higher prevalence rates of severe cardiac siderosis [19,20,21,22]. Heart failure occurs in only one patient with severe cardiac siderosis. No patient died in our five-year retrospective cohort study.

In our study, initial cardiac T2* MRI values below 25 ms resulted in adjusted iron chelation therapy in 16 cases, with cardiac T2* MRI values marginally below 25 ms in five patients. The cardiac MRI results improved as the iron chelation therapy was intensified, and no confirmed cases of heart failure occurred.

In severe cardiac siderosis cases, the impaired ventricular function was reported in up to 62 percent of patients [19,20,21,22,23]. According to a previous study, the systolic function has low sensitivity for detecting elevated myocardial iron. The study’s authors found that 18% of their patients had elevated myocardial iron with a normal ejection fraction [20]. Left ventricular function deteriorates as myocardial T2* decreases and is followed by left ventricular dilation and hypertrophy. Three patients with severe cardiac siderosis in the present study were asymptomatic and had a normal ejection fraction. Clinically significant myocardial complications, such as heart failure and life-threatening ventricular arrhythmias, are more likely to occur in these patients. These complications could be avoided if their chelation regimen was optimized [8,20,21,22]. The decrease in heart failure rates in our patients with severe cardiac siderosis may be attributed to strict monitoring, aggressive treatment, and chelation optimization.

No correlation was found between cardiac T2* and liver iron concentration in the present study. This has been previously described in various studies, with a significant disassociation between liver and heart iron values [4,5,6,12]. Since there is no correlation, cardiac T2* MRI cannot be predicted from LIC and vice versa. This was demonstrated using organ-specific iron uptake/release mechanisms. Furthermore, there are variations in iron elimination; for example, chelation therapy will eliminate iron from the liver more rapidly than from the heart, allowing liver iron to normalize while myocardial iron remains elevated [12,24,25].

During the five-year follow-up period, mean LVEF increased and improved cardiac T2* MRI in our study. Nevertheless, no substantial relationship was found between the percent of improvement in cardiac T2* and the percent shift in LVEF. This could be attributed to the limited sample size and the fact that most participants had normal LVEF.

The present study’s limitation is the small number of severe cardiac siderosis participants and the low incidence of heart failure patients, and there were differences in length of time between MRI scans.

## 5. Conclusions

The study validates the current practice of using the cardiac T2* MRI to adjust iron chelation therapy in patients with cardiac siderosis by revealing that patients’ cardiac T2* MRI improved over time. Since serum ferritin did not appear to be an effective indicator of iron overload in these patient populations, it is essential to make cardiac T2* MRI iron overload assessments more available to patients, and the results could be used to encourage patients to take their chelation therapy and customize it to reduce their iron overload and hence reduce morbidity and mortality.

## Figures and Tables

**Table 1:** Baseline patients characteristics with normal, marginal, mild to moderate, and severe cardiac siderosis.

Patient Characteristics	Normal (n = 98)	Marginal (n = 5)	Mild to Moderate (n = 12)	Severe (n = 4)	*p*-Value
Mean age (years)	27.9 ± 13.1	28.1 ± 6.8	37.7 ± 11.6	37.8 ± 9.7	0.14
Male gender, n (%)	54 (55.1)	3 (60)	7 (58.3)	3 (75)	0.83
Thalassemia type					
Homozygous beta thalassemia, n (%)	43 (43.9)	2 (40)	5 (41.7)	2 (50)	0.88
Beta thalassemia/HbE, n (%)	55 (56.1)	3 (60)	7 (58.3)	2 (50)	0.81
History of splenectomy, n (%)	36 (36.7)	3 (60)	3 (25)	4 (100)	0.01 *
Mean pre-transfusion hemoglobin level (g/dl)	9.5 ± 0.3	9.4 ± 0.3	9.4 ± 0.4	9.4 ± 0.4	0.84
Mean baseline ferritin level (mg/dl)	2523.8 ± 1561.4	4741.9 ± 2184.6	3535.4 ± 2113.6	4189.1 ± 2172.6	0.06
Median baseline LIC (mg/g dry weight)	19.2 ± 5.5	25.3 ± 7.7	21.6 ± 13.3	23.9 ± 6.5	0.16
Mean LVEF (%)	63.6 ± 14.5	59.5 ± 13.2	61.9 ± 13.6	58.3 ± 11.1	0.69

Value is mean ± SD; * Statistically significant at *p*-value < 0.05 determined by repeated measure ANOVA with Greenhouse–Geisser.

**Table 2:** Baseline and mean cardiac T2*at one year, three years, and five years in 4 groups.

Cardiac T2 *	Baseline	One Year	Three Year	Five Year	F	*p*-Value
Normal (n = 98)	43.1 ± 7.2	42.2 ± 6.6	43.4 ± 6.1	43.6 ± 5.6	194	0.59
Marginal (n = 5)	23.2 ± 1.2	31.8 ± 5.8	31.6 ± 3.9	33.5 ± 1.6	8	<0.001 *
Mild to moderate (n = 12)	15.8 ± 3.4	29.2 ± 6.4	34.2 ± 4.6	38.9 ± 5.5	22	<0.001 *
Severe (n = 4)	8.5 ± 1.5	17.1 ± 3.4	30.3 ± 6.1	33.9 ± 1.9	6	<0.001 *

Value is mean ± SD; * Statistically significant at *p*-value < 0.05 determined by repeated measure ANOVA with Greenhouse–Geisser.

**Table 3:** Comparison between the parameters at baseline and one year, three years, and five years.

Parameters	Time				F	*p*-Value
	Baseline	One Year	Three Year	Five Year		
T2 *	16.24 ± 5.63	27.57 ± 7.72	32.86 ± 4.77	36.62 ± 4.99	68.126	<0.001 *
LIC	22.86 ± 10.92	14.33 ± 5.25	10.71 ± 5.33	5.43 ± 2.77	40.515	<0.001 *
Ferritin	4175 ± 2120	3034 ± 1438	2425 ± 901	1546 ± 691	17.953	<0.001 *
LVEF	57.43 ± 3.04	59.38 ± 4.48	60.19 ± 3.93	60.57 ± 3.41	16.789	0.61

Value is mean ± SD; * Statistically significant at *p*-value < 0.05 determined by repeated measure ANOVA with Greenhouse–Geisser.

**Table 4:** Iron chelation therapy and volume of red cell transfusion of thalassemia patients with and without cardiac siderosis.

	Patients with Cardiac Siderosis (n = 21)	Patients without Cardiac Siderosis (n = 98)	*p*-Value
Iron chelation therapy			
DFO, n (%)	11 (52.4)	28 (28.6)	0.36
DFX, n (%)	4 (19)	20 (20.4)	0.89
DFP, n (%)	2 (9.6)	49 (50)	0.0007 *
Combination therapy, n (%)	4 (19)	1 (1)	0.0002 *
Volume of red cell transfusion (unit/year)	19.6 ± 6.9	11.1 ± 7.8	<0.0001 *

DFO: Desferrioxamine, DFX: Deferasirox, DFP: Deferiprone; * Statistically significant at *p*-value < 0.05.
